# Sol-Gel ceramic glazes with photocatalytic activity

**DOI:** 10.1007/s10971-022-05787-z

**Published:** 2022-04-23

**Authors:** G. Monrós, M. Llusar, J. Badenes, R. Galindo

**Affiliations:** grid.9612.c 0000 0001 1957 9153 Dpt. Química Inorgànica i Orgànica, Universitat Jaume I, Castellón, Spain

**Keywords:** Ceramic glaze, Sol-Gel, Photocatalysis

## Abstract

A frit is a glassy ceramic composition that has been fused, quenched, and granulated. A single frit or a mixture of frits and ceramic materials forms a ceramic glaze. The purpose of this pre-fusion is to render any soluble and/or toxic components insoluble by rendering it inert in a glassy composition with silica and other added oxides. The ceramic glaze dispersed in water (ceramic slip) is deposited on a ceramic body and fired for waterproofing and aesthetic purposes. Multicomponent frits (zinc-potassium borosilicate system) with similar behavior to conventional ceramic frits for single-firing ceramic glazes (“monoporosa” glazes fired at 1080 °C) were prepared by Sol-Gel methods (monophasic and polyphasic gels) avoiding the pre-fusion and characterized as photocatalytic agents (showing high degradation activity on Orange II). The effect of doping with bandgap modifiers (V_2_O_5_, Sb_2_O_5_ and SnO_2_) and also with devitrification agents (ZrO_2_ to crystallize zircon, Al_2_O_3_ to anorthite, Mo_2_O_3_ to powellite and ZnO to gahnite ZnAl_2_O_4_) were analyzed.

**Figure:**
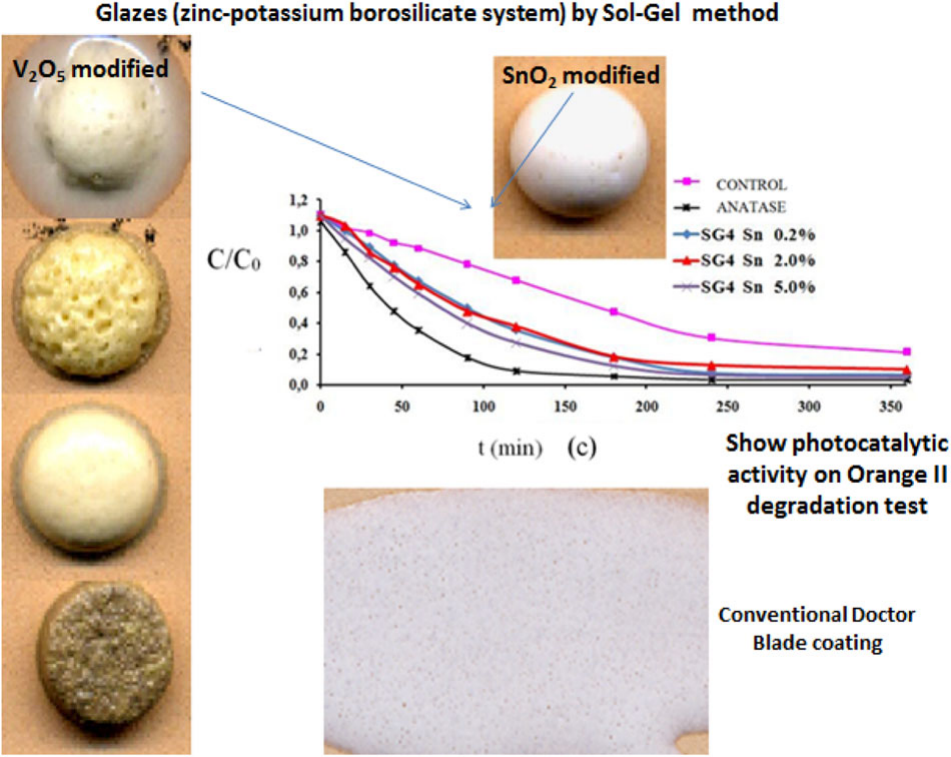
Multicomponent frits (zinc-potassium borosilicate system) with similar behavior to conventional ceramic frits for single-firing glazes (1080 °C) prepared by Sol-Gel methods (monophasic and polyphasic gels), without pre-fusion, shows photocatalytic activity.

Multicomponent frits (zinc-potassium borosilicate system) with similar behavior to conventional ceramic frits for single-firing glazes (1080 °C) prepared by Sol-Gel methods (monophasic and polyphasic gels), without pre-fusion, shows photocatalytic activity.

## Highlights

- Multicomponent frits (zinc-potassium borosilicate system) with similar behavior to conventional ceramic frits were prepared by Sol-Gel methods without pre-fusion.
- Zinc and potassium borosilicate-based glaze, modified with alumina and calcium, is considered the optimum for conventional applications, and shows a moderate photoactivity with a half-life period of 77 min.
- Doping with bandgap modifiers enhances the photoactivity; V_2_O_5_ or SnO_2_ show good photoactivity in the 5 w% sample (t_1/2_ = 55 and 58 min, respectively).
- Doping with devitrification agents (ZrO_2_ to crystallize zircon, Al_2_O_3_ to anorthite, Mo_2_O_3_ to powellite and ZnO to gahnite ZnAl_2_O_4_) shows that the photocatalytic activity is low, similar or lower than the SG4 zinc-potassium borosilicate parent glaze.

## Introduction

### Ceramic glazes: characteristics and preparation

In ceramics, glazes are prepared by fusion generating materials generally referred to as frits. To obtain a frit, raw materials such as oxides are used to obtain said composition, considering the losses during firing and decomposition. The raw materials are mixed in a mill and melted in rotary or continuous furnaces at 1500–1600 °C. The molten material is rapidly cooled (quenching) over water or on air-cooled rollers to obtain a glass frit in the form of granules or flakes.

The glasses are formulated using the so-called Seger’s formula [1] which is a molar ratio of basic oxides or fluxes (which are indicated in the column on the left), amphoteric oxides (indicated in the center) and acidic oxides (formers indicated in column on the right). Below are the typical formulations of common glasses.

i) Window glass (soda lime): fusible glass (T_g_ 600 °C and softening at 700 °C), perfect for flat glass.
  $$\begin{array}{*{20}{c}} {\left. \begin{array}{l}0.6\,{{{\mathrm{Na}}}}_2{{{\mathrm{O}}}}\\ 0.4\,{{{\mathrm{CaO}}}}\end{array} \right\}} & {0.05\,{{{\mathrm{Al}}}}_2{{{\mathrm{O}}}}_3} & {\left\{ \begin{array}{l}\\ 3.3{{{\mathrm{SiO}}}}_2.\end{array} \right.} \end{array}$$

ii) Borosilicate glasses: infusible at flame and resistant to thermal shock.
  $$\begin{array}{l}\begin{array}{*{20}{c}} \begin{array}{l}{{{\mathrm{JENA}}}}\\ \left. \begin{array}{l}0.5\,{{{\mathrm{Na}}}}_2{{{\mathrm{O}}}}\\ 0.5\,{{{\mathrm{Cao}}}}\end{array} \right\}\end{array} & {1\,{{{\mathrm{Al}}}}_2{{{\mathrm{O}}}}_3} & {\left\{ \begin{array}{l}3\,{{{\mathrm{SiO}}}}_2\\ 0.5\,{{{\mathrm{B}}}}_2{{{\mathrm{O}}}}_3.\end{array} \right.} \end{array}\\ \begin{array}{*{20}{c}} \begin{array}{l}{{{\mathrm{PYREX}}}}\\ \left. \begin{array}{l}0.4\,{{{\mathrm{Na}}}}_2{{{\mathrm{O}}}}\\ {{{\mathrm{0}}}}{{{\mathrm{.6}}}}\,{{{\mathrm{Cao}}}}\end{array} \right\}\end{array} & {0.1\,{{{\mathrm{Al}}}}_2{{{\mathrm{O}}}}_3} & {\left\{ \begin{array}{l}8\,{{{\mathrm{SiO}}}}_2\\ 1\,{{{\mathrm{B}}}}_2{{{\mathrm{O}}}}_3.\end{array} \right.} \end{array}\end{array}$$

In the enameling process, the glaze with the appropriate additives (pigments, mill additions, glue, etc) are ground in a ball mill in water medium to form a slip or suspension of the material of suitable viscosity to be deposited by immersion, Doctor Blade or spraying on a ceramic support.

Historically, glazes have been based on wood ash (completely burned in a kiln that results in a 1 wt% mixture of fine particles of CaCO_3_, K_2_CO_3_ and phosphates), following the invention of glass around 1500 BC in the Middle East and Egypt. At around 100 BC, lead-glazing was widespread in the known world (lead glazed earthenware was probably made in China during the Warring States Period, 475–221 BC). Subsequently the lead-silica was modified with 2–5wt% of tin oxide leading to the tin-opacified glaze; a white, glossy and opaque glaze discovered in Iraq (8th century), developed by the Islamic potters and prevalent in the ceramics industry until the 20th century [2].

In around 1914, tin was replaced by zircon as an opacifier due to the high increase in the price of cassiterite (SnO_2_), likewise lead was replaced by boron and alkaline fluxes due to the high toxicity of lead. Previously, in order to avoid the leaching of lead and other hazardous ions from glazes, the raw materials were “fritted”: the composition was fused and quenched to form a glass, and then granulated. The purpose of this pre-fusion was to render any soluble and/ or toxic components insoluble by causing them to combine with silica and other added oxides [3].

### Sol-Gel glazes

Sol-Gel processes were developed during the second half of last century as an alternative for the preparation of glasses and ceramics at low temperature (the first International Conference on Glasses and Glass Ceramics Obtained from Gels was held in Padova, Italy in 1981) resulting in a pioneering nanotechnology because all Sol-Gel products may contain nanoparticles or are nanocomposites as pointed out by Sumio Sakka, the first editor-in-chief of the Journal of Sol-Gel Science and Technology in 1993 [4].

**Fig. 1:**
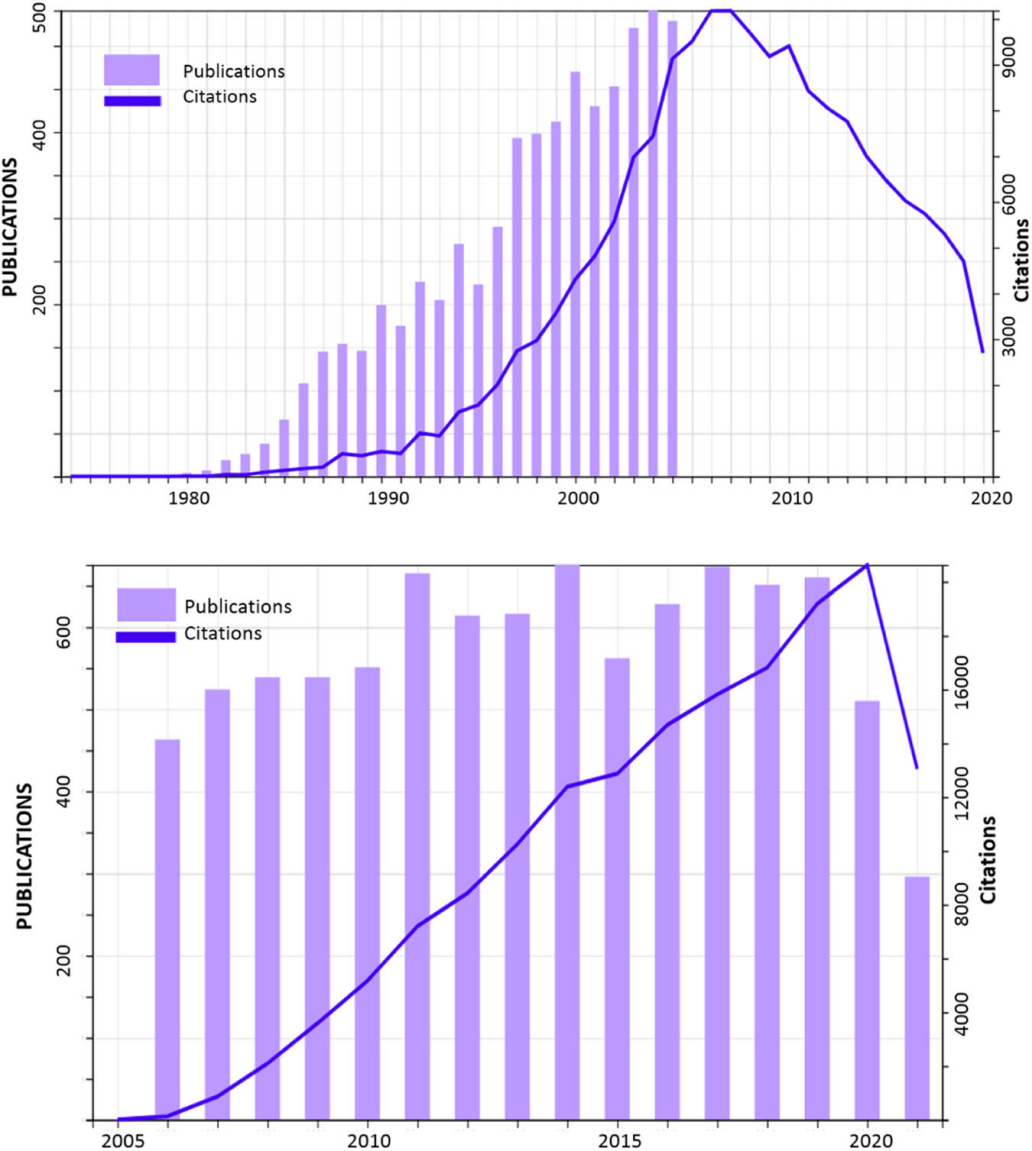
Evolution of publications and citations for 1974–2005 and 2006–2021 (Web of Science at http://www.webofknowledge.com), retrieved 10 August 2021)

If a simple search is carried out in databases such as Web of Science (WOS) using the keyword “Sol-Gel glass” there are 6450 entries referring to materials science between 1974 and 2005; in the period from 2006–2021 there are 9166 entries. Figure 1 shows the evolution of publications and citations for both periods. It is observed that the number of publications grows exponentially in the period 1974–2005 (with the strong start in 1982 after the Padova congress and in 1995 after the first issue of the Journal of Sol-Gel Science and Technology) stabilizing at around 550 per year until the strong decrease in 2020 associated with the general crisis of the COVID-19 pandemic. First developed in the 1960s, Sol-Gel processes had the intended purpose of producing bulk glasses at low temperatures, below 1000 °C [5–8]. Sol-Gel techniques greatly differed from standard practices and energy-intensive melting methods which typically involve temperatures well over 1400 °C in furnaces [9]. Years later, the rising popularity of optical fibers stimulated research into the production of silica glass preforms, from which optical glass fibers are drawn, via the sol-gel method [10]. Thus, with the Sol-Gel process, new glass compositions that could not be achieved with melt-quenching were made possible [11–17].

Between the main applications of Sol-Gel in the glass field are the optical glasses [18–20] and bioglasses [21–23]. Although ceramic glazes are true glass, glazes are meant to be applied as a thin coating on the surface of ceramic substrates and usually are multicomponent materials. Initially, Sol-Gel glasses involved only a small number of components [6, 10] and were based on conventional alkoxides of glass formers (acids in Seger’s formula) such as Si, Sn, Ti, Zr; [5, 6] later, they were progressively applied to multicomponent systems [16, 20, 24], with improved properties compared to the conventional melt quench method. For example, Larry Hench’s 45S5 Bioglass was the first artificial material that was found to form a chemical bond with bone, launching the field of bioactive ceramics. Hench used the conventional melt quench method for the 45S5 Bioglass synthesis [21]. However, calcium phosphates such as tricalcium phosphate and synthetic hydroxyapatite are more widely used clinically due, among other reasons, to the fact that it is difficult to produce porous bioactive glass templates for bone regeneration from Bioglass 45S5 because it crystallizes during sintering. In order to combine the properties of apatite with those of bioglass, Sol-Gel glass ceramics have been developed that devitrify apatite in the SiO_2_–CaO–P_2_O_5_–MgO quaternary glass system. For example, Kokubo [23] prepared MgO-CaO-SiO_2_-P_2_O_5_ glass that, by heat treatment, gave a glass ceramic containing crystalline apatite (Ca_10_(PO_4_)_6_O,F_2_)) and β-wollastonite (CaO.SiO_2_) in an MgO-CaO-SiO_2_ glassy matrix. Balamurugan et al. [25] prepared bioglass in the CaO-P_2_O_5_-SiO_2_-ZnO system, introducing ZnO in the composition by the Sol-Gel technique.

### Photocatalysis and glazes

The photocatalytic oxidation of aqueous and gaseous contaminants has been extensively studied since the research of photocatalytic water splitting on TiO_2_ electrodes was first conducted in 1972. It has drawn considerable academic interest as a very attractive, nonselective, room-temperature process for the degradation of organic pollutants, based on the illumination of a semiconductor that give photoexcited electrons and holes that will decompose contaminants adsorbed on the photocatalyst surface. TiO_2_ (usually in the anatase polymorph) is widely used in environmental applications because of its physical and chemical stability, lower cost, non-toxicity, and resistance to corrosion. For the practice of photocatalytic reactions, it is necessary to have light of a sufficient energy that exceeds the TiO2 band gap energy (Eg: for anatase, 3.2 eV; for rutile, 3.02 eV, corresponding to absorbance thresholds of 380 and 410 nm, respectively). However, the absorbance wavelength of anatase does not conform to the solar spectrum region; the solar energy of about 3.0 eV (λ ≤ 410 nm) is >5%. Thus, it cannot effectively utilize visible light [26].

The doping of TiO2 with nonmetals, such as C, N, S, P shows significant improvement in causing photosensitization in the visible region. For these anion-doped TiO2 photocatalysts, these species substitute for oxygen in the TiO2 lattice and lead to a band gap decrease, resulting in high visible spectrum absorbance due to the contribution of their p orbitals [27, 28]. P-doped TiO2 was prepared by a simple modified Sol-Gel method with hypophosphorous acid as a precursor, and it was found that P-TiO2 had a significantly increased surface area which consequently provided a higher content of surface hydroxyl groups, thus elevating photocatalytic activity [27, 28]. On the other hand, doping with transition metals, such as Fe, Pd, and Pt, TiO2 could lead to the absorbance of the photocatalyst shifting into the visible range but trigger a considerable decrease in photocatalytic activity [27, 29].

Reddam et al. [30] studied the photocatalytic degradation of the azoic dye “Orange II” using Mn-, Cu- and Fe-modified titania. The catalysts prepared by two different methods (Sol–Gel and an impregnation of commercial titania (Degussa P25)), were calcined at 400 °C. The catalysts prepared by the Sol–Gel method exhibit a larger surface area but lower photoactivity due to the presence of both anatase and rutile phases in the impregnated samples that facilitate transfer of the photogenerated electron and prevented electron–hole recombination. Although the presence of metallic cations in solid solution enhance the formation of electrons–hole pairs, at a high doping content, a large number of structural defects are induced that act as recombination centers and Sol–Gel samples show lower photoactivity than impregnated samples, except in the case of Fe-modified samples that with low bandgap compensates the electron–hole recombination. The authors conclude that to obtain a highly active catalyst, it is necessary to optimize four properties of the samples: the rutile phase content, the dopant content, the chemisorbed oxygen content and the surface area [30].

Usually, it is reported that TiO2 might be deactivated after a period of use and that the intermediates and final products produced from photocatalytic oxidation, and which cover the catalytic surface, are responsible for deactivation. For real-life application the photocatalysis should be reversible with respective to deactivation caused by oxidized intermediates or absorbed byproducts on the surface of these catalysts [26]. There are three methods used to regenerate deactivated photocatalyst: thermal regeneration at around 400–450 °C under air flow [31], photocatalytic regeneration (e.g. under the UV lamp used with oxygen flow [32]) and regeneration through washing.

Sol-Gel glass powders containing titania can exhibit photocatalytic activity. For example, Gargori et al. [33] prepared glasses with SiO_2_-CaO-ZnO-B_2_O_3_-K_2_O-Al_2_O_3_ composition modified by the addition of titania (3, 5, 12 and 20 w%) by Sol-Gel processing their photocatalytic activity for the degradation of orange II dye was studied. XRD and TEM results indicated that the nanostructured system was amorphous. From UV-Vis-NIR results the calculated band gap was around 3.5 eV for all samples. The photoactivity of the powders depended on the amount of titania in the glass composition and the surface area of the samples. The sample with highest surface area and lowest addition of titania (3 w%) shows similar activity to commercial anatase used as reference.

Conventional glazes can also exhibit photocatalytic activity. Ruiz et al. [34] demonstrated the capacity of some glazes that devitrify some crystalline phases to develop photocatalytic activity that degrades Orange II organic colorant. Glazes with crystalline phases such as anorthite, cassiterite and zircon improve photocatalytic degradability with respect to the initial standard transparent glaze. When the devitrified zirconium silicate content increased, the half-life period decreased, and the photochemical degradability of Orange II therefore improved. Although the band gap energy (E_g_) values in the glazes are in the range 3.5–3.9 eV (higher than anatase used as reference E_g_ = 3.23 eV), this condition is necessary but not sufficient to develop glaze photocatalytic degradability. Indeed, the evolution of the microstructure plays an important role in the photocatalytic properties of a glaze because, for similar values of E_g_, an increase in the concentration of the devitrified zircon phase, as well as in crystal size, results in an improvement in the photocatalytic activity.

The coating of glazes by films of selected wide band semiconductors also show photocatalytic activity; Ruiz et al. [34] determined the optimum composition for photocatalytic activity of films deposited using the usual screen printing of titanium isopropoxide dissolved in polyol vehicles such as ethylene glycol. The second firing was conducted at 790 °C, yielding layers of lusters with different TiO_2_ contents. In order to confirm the crystallization of anatase, grazing incidence X-ray diffraction analysis was carried out. The optimum composition, from a photocatalytic viewpoint (minimum half time t_1/2_), corresponded to a TiO_2_ content of 4.0 w% (t_1/2_ = 34.5 min) with a photocatalytic activity equal to or greater than that of the anatase powder used as reference (t_1/2_ = 42.6 min). Likewise, Cerro et al. [35] prepared coatings of silica, bismuth oxide, zirconia and anatase on a parent glass in the SiO_2_–CaO–ZnO–B_2_O_3_–K_2_O–Al_2_O_3_ system (with additions of ZrO_2_), deposited and processed by single firing (1085 °C). The photocatalytic activity of the samples determined by degradation of Orange II showed that a first order reaction according to the Langmuir-Hinshelwood model is followed. From UV-Vis-NIR results, the band gap was around 3.5 eV for the parent glass and that with a silica coating, and slightly lower for the other coatings. The needle-shaped microstructure of the parent glass (associated with zircon crystallization) shows the best photocatalytic results in agreement with the literature [34] (t_1/2_ = 103 min compared to 38 min for P25 Degussa used as reference). The preserved zircon microstructure can explain the relatively high results for the silica coating (t_1/2_ = 123 min), which unexpectedly showed better results than the anatase or tetragonal zirconia coatings. Finally, the interaction with the parent glass can explain the relatively high results of the bismuth oxide (t_1/2_ = 183 min).

In this paper multicomponent frits (zinc-potassium borosilicate system) with similar behavior to conventional ceramic frits for single-firing ceramic glazes were prepared by Sol-Gel methods (monophasic and polyphasic gels) avoiding the pre-fusion step and characterized as photocatalytic agents on Orange II. The effect of doping with bandgap modifiers (V_2_O_5_, Sb_2_O_5_ and SnO_2_) and also with devitrification agents (ZrO_2_ to crystallize zircon, Al_2_O_3_ to anorthite, Mo_2_O_3_ to powellite and ZnO to gahnite ZnAl_2_O_4_) are analyzed.

## Experimental

### Preparation of glazes

**Fig. 2:**
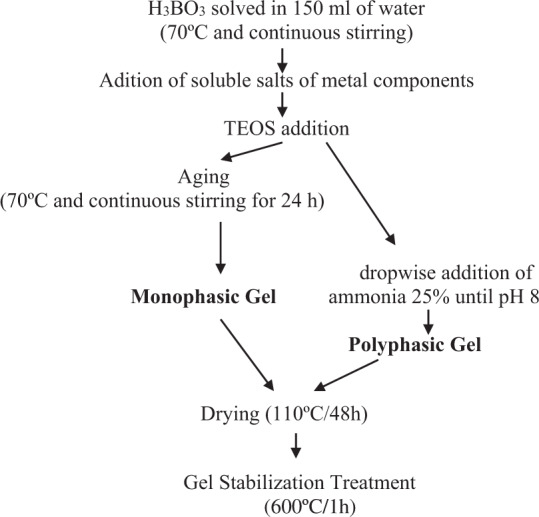
Monophasic and polyphasic gels preparation.

All precursors were supplied by PANREAC SA (analysis quality). Four formulations of glazes subsequently referred to as SG1, SG2, SG3, and SG4 were prepared (Table 1). All glazes were obtained by a monophasic Sol-Gel process by hydrolysis-condensation of TEOS and soluble salts in water and a polyphasic or colloidal precipitation by destabilizing the solution with ammonia (Fig. 2) for a quantity of final product of 20 g [36].

**Table 1:** Chemical composition of glazes (w%) and stoichiometry of Seger for SG4 sample

	SG1 parent glaze	SG2 calcic	SG3 aluminous	SG4 calcic-aluminous
SiO_2_	50	50	50	50
CaO	-	18	-	18
K_2_O	4	4	4	4
Al_2_O_3_	-	-	8	8
ZnO	12	12	12	12
B_2_O_3_	8	8	8	8

$$\begin{array}{l}\quad \quad {{{\mathrm{SG}}}}4\left( {{{{\mathrm{SEGER}}}}^\prime {{{\mathrm{s}}}}\,{{{\mathrm{FORMULA}}}}} \right)\\ \begin{array}{*{20}{c}} {\left. \begin{array}{l}0.22\,{{{\mathrm{K}}}}_2{{{\mathrm{O}}}}\\ 0.78\,{{{\mathrm{ZnO}}}}\end{array} \right\}} & {0.39\,{{{\mathrm{Al}}}}_2{{{\mathrm{O}}}}_3} & {\left\{ \begin{array}{l}6.27\,{{{\mathrm{SiO}}}}_2\\ 0.58\,{{{\mathrm{B}}}}_2{{{\mathrm{O}}}}_3.\end{array} \right.} \end{array}\end{array}$$

As shown in Fig. 2, boric acid H_3_BO_3_ was dissolved firstly (pH = 7), then the corresponding soluble nitrate salts of the component metals (Zn(NO_3_)_2_.6H_2_O, Al(NO_3_)_3_.9H_2_O, KNO_3_, Ca(NO_3_).4H_2_O) were dissolved. No precipitation was observed. After hydrolysis, the monophasic gel samples produced homogeneous and transparent gels. The pH of the SG1 solution was 4.5 prior to gelation; for the other monophasic systems, the solution pH prior to gelation was pH = 2.

### Samples characterization

X-ray diffraction (XRD) was carried out on a Siemens D5000 diffractometer using Cu K_α_ radiation (10–70 °2θ range, scan rate 0.02 °2θ/s, 4 s per step and 40 kV and 20 mA conditions).

UV-Vis-NIR spectra of fired powder samples and also of the applications of the pigments samples were collected using a Jasco V670 spectrometer using the diffuse reflectance technique. The band gap energy of semiconductors was calculated from Tauc plots using the UV-Vis-NIR spectra plotted using the Kubelka-Munk model [37, 38].

The Tauc method is based on the assumption that the energy-dependent absorption coefficient, α, is given by:

1 $$\left( {\alpha \,h\nu } \right)^{\curlyvee} = {{{\mathrm{B}}}}\left( {{{{\mathrm{h}}}}\nu -{{{\mathrm{E}}}}_{{{\mathrm{g}}}}} \right)$$

where h is the Planck constant, ν is the photon’s frequency, E_g_ is the band gap energy, and B is a constant (which is determined by the index of refraction, electron, and hole effective masses; however, it is usually taken as 1 for amorphous materials). The absorption coefficient α is calculated from the absorbance data following the Beer-Lambert law. The important term is the exponent γ, which denotes the nature of the electronic transition. When γ = 2 it is a direct allowed transition, and when it is equal to 1/2, it is an indirect allowed transition. A semiconductor has a direct band gap when the valence band maximum and the conduction band minimum are at the same point of the Brillouin zone; in these materials, a photon can directly excite an electron from the valence to the conduction band if the energy is at or above the gap energy.

The photocatalytic tests were carried out using a dispersion of 500 mg/l of powder added to a solution 6.0 × 10^−6 ^M of orange II in a pH 7.42 phosphate buffer medium (NaH_2_PO_4_·H_2_O, 3.31 g, and Na_2_HPO_4_·7H_2_O, 33.77, g dissolved in l.00 l of water) in all performed tests. The dye solution was analyzed by UV-Vis-NIR diffuse reflectance to determinate λ_max_ (485 nm). The UV irradiation source was a 125 W mercury lamp emitting in the range 254–365 nm. The suspension was first stirred in the dark for 15 min to reach equilibrium sorption of the dye. Aliquot samples were taken every 15 min to measure the change in the dye concentration, after sedimentation of the catalyst. Blank experiments with Orange II solution and without catalyst, were conducted before the photocatalytic experiments (CONTROL). Likewise commercial anatase P25 from Degussa was used as reference to compare its photocatalytic activity with the prepared samples (ANATASE) [39]. The resulting photodegradation curves were analyzed following the Langmuir-Hinshelwood model [39].

The thermal performance of the glazes was studied by Doctor Blade deposition of the glaze slurry in water on red stoneware using a single fire cycle (monoporosa cycle: room temperature −800 °C in 5 min, 800–1080 °C in 25 min, soaking at 1080 °C for 5 min, cooling from 1080 to 600 °C in 20 min and final free cooling). On the other hand, thermal evolution of fusion buttons were analyzed by preparing buttons with 0.5 g of sieved powders (116 µm) pressed in a manual press at 45 bar.

Microstructure characterization of fired Doctor Blade deposition was carried out by Scanning Electron Microscopy (SEM) using a JEOL 7001 F electron microscope (following conventional preparation and imaging techniques).

## Results and discussion

The glazes that will be discussed below are explicitly identified as direct (D) or indirect (I) as described in Tables together its calculated bandgap. In the case of indirect case, as in the original work of Jan Tauc for amorphous germanium, the spectrum shows a tail associated to localized states at lower energies (Urbach tail (38)) and proposed an extrapolation to find the optical bandgap of these crystalline-like states.

**Fig. 3:**
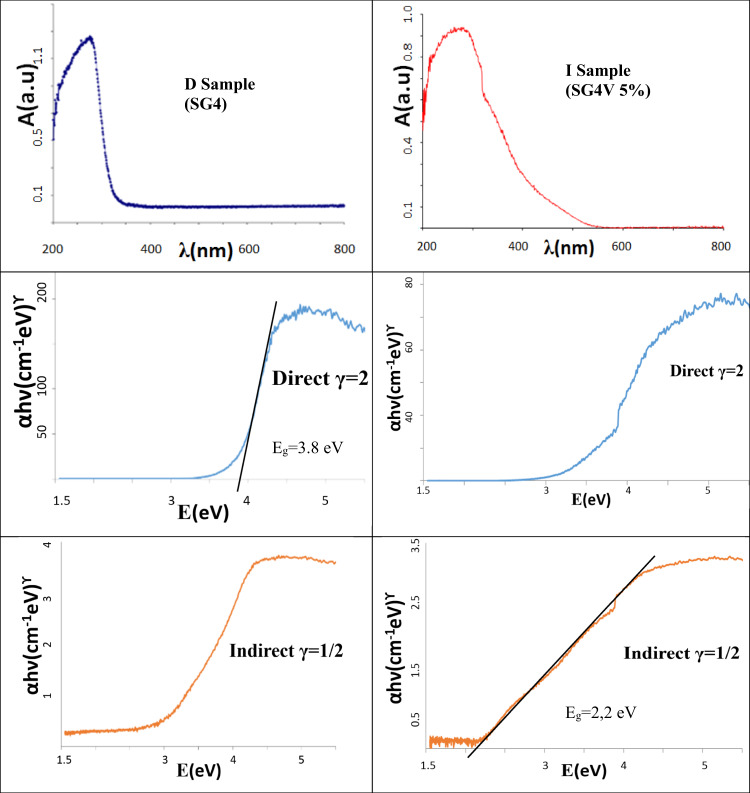
UV-Vis-NIR spectra and the associated Tauc plots of two representative samples discussed below considered direct (D, γ = 2) or indirect (I, γ = 1/2) semiconductor.

Figure 3 shows the UV-Vis-NIR spectra and the associated Tauc plots of two representative samples below discussed considered direct (D, γ = 1/2) or indirect (I, γ = 2) semiconductor; the UV-Vis-NIR spectrum for the direct semiconductor shows only one band and for indirect case the spectrum shows a tail associated to localized states at lower energies with an extrapolated bandgap of 3.8 and 2.2 eV, respectively.

### Multicomponent frits prepared by Sol-Gel method (zinc-potassium borosilicate system)

Although an attempt was made to glaze both the raw gels and the dry powders (not shown), after mixing with the appropriate amount of water to give a stable and manageable slip by Doctor Blade technique, the depositions on a porous ceramic support cracked, due to the excessive presence of salts, crusting and low rates of coverage when fired with a single fire cycle (1080 °C).

**Fig. 4:**
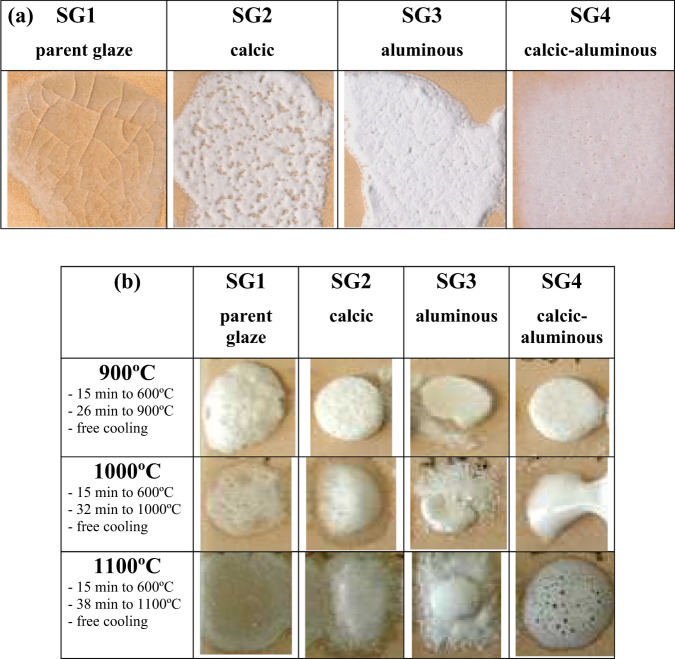
Depositions of monophasic gels stabilized at 600 °C: (**a**) films deposited using the Doctor Blade technique 6 mm on a red porous support and fired in a single fire cycle (1080 °C), (**b**) evolution of the melting buttons with temperature.

Figure 4a shows the glaze layers of monophasic gels stabilized at 600 °C (Fig. 2) deposited by the Doctor Blade technique with a thickness of 6 mm on a red porous support and fired with a single firing cycle (1080 °C). The SG1 zinc potassium borosilicate parent glaze appears transparent, with a cracked appearance, given its low thermal expansion coefficient; the addition of calcium or alumina, or both, produced more refractory white opaque glazes. Indeed, the evolution of the fusion buttons with temperature in Fig. 4b. confirms the transparency of the parent glaze that melts with low viscosity. Samples SG2 and SG3 are more refractory and good melting is not observed. In contrast the calcium-aluminous glaze SG4, generates a viscous fluid at 1000 °C that exhibits degassing observable both at 1000 °C and in single fire cycle (1080 °C) in Fig. 4a.

**Fig. 5:**
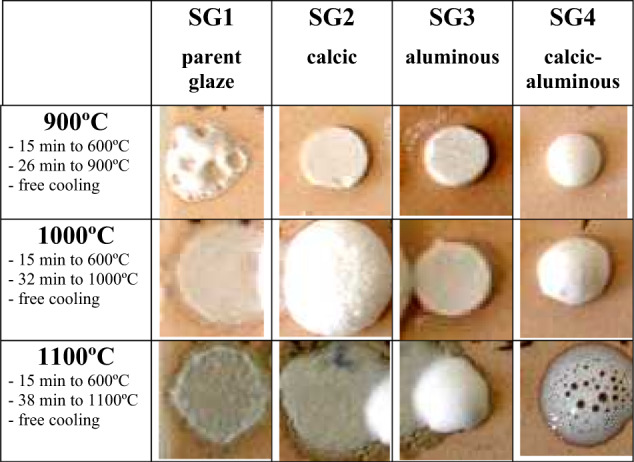
Evolution of the melting buttons with the temperature of the polyphasic samples stabilized materials at 600 °C.

Figure 5 shows the evolution of the melting buttons with the temperature of the stabilized materials at 600 °C. Although vitrification is evident in all samples, the four samples show opaque white vitrification. A similar behavior is observed to the monophasic samples of Fig. 4b in the case of sample SG4, which degasses at 1100 °C. The melting and transparency of the parent glaze (SG1) and the more refractory character of samples SG2 and SG3 are also evident. The X-ray diffraction of all the glazes stabilized at 600 °C (not shown) is typical of a siliceous glass with the amorphous halo centered at 27° 2θ.

Although the comparison of Fig. 4b and Fig. 5 shows that vitrification develops more homogeneously in the case of monophasic samples, the difference is not considered significant, and for its simplicity the polyphasic process results are more interesting. Likewise, the SG4 glaze, which produces an adequate opaque vitrification in a single firing cycle, is considered the optimum for conventional applications.

**Fig. 6:**
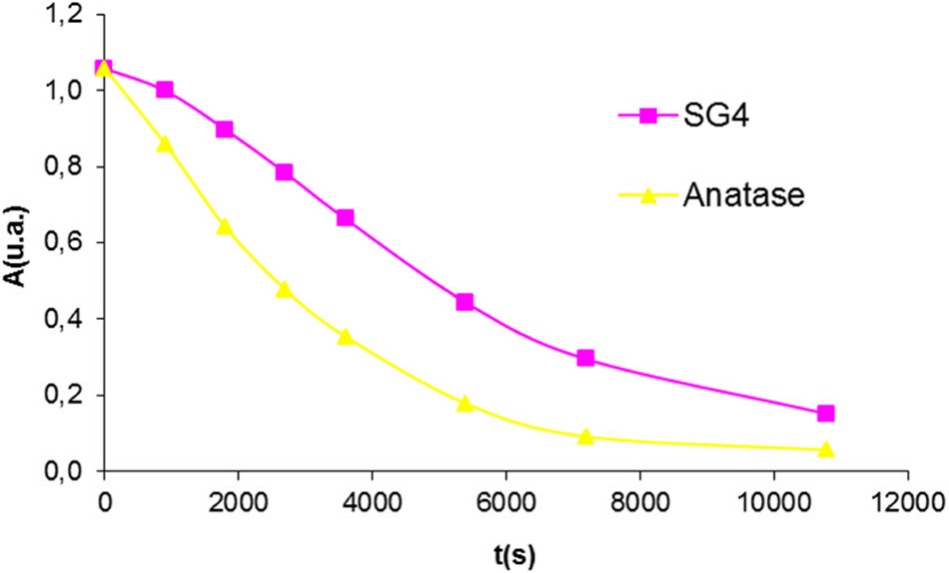
Orange II photodegradation test of SG4 compared to the reference anatase.

The Orange II photodegradation test of SG4 polyphasic sample stabilized at 600 °C compared to the reference anatase, whose absorbance-time curves are presented in Fig. 6 shows that the SG4 glaze has lower photoactivity than the reference anatase. Using the Langmuir-Hinshelwood method, the estimated half-life of the glaze is t_1/2_ = 77 min compared to 42 min for the anatase reference.

### Effect of doping with bandgap modifiers (V_2_O_5_, Sb_2_O_5_ and SnO_2_)

Modifications in the band gap of semiconductor or insulating materials can be induced by doping [40, 41]. This section discusses the doping of the previously described SG4 glaze with different agents capable of modifying the band gap of the glaze.

#### Effect of doping with vanadium or niobium

Vanadium pentoxide is a ferroelectric n-type semiconductor with a Curie temperature of 250 °C and a band gap of 1 eV, at 25 °C; the concentration of negative carriers is 1.6 × 10^13 ^cm^−3^ with a mobility of 400 cm^2^V^−1^ [42, 43]. On the other hand, Nb_2_O_5_ is an insulator with a conductivity of 3 × 10^−6^ S/cm in monocrystalline form. However, polycrystalline Nb_2_O_5_ exhibits a defect structure with oxygen vacancies, so that Nb_2_O_4.978_ has a conductivity of 3 × 10^3 ^S/cm. Its conduction band is made up of the 3d orbitals of Nb and the valence band of the 2p orbitals of oxygen [44]. The effect of doping on rutile titanium oxide has been studied. A second crystalline phase is observed along with rutile when the dopant content exceeds 0.7 mol%. Such doping also helps to sinter the rutile and doping induces defects at the borders of rutile grain, reducing its electrical resistance at high temperatures [45–47].

Phosphate glasses with unusually high vanadium contents have been studied in the literature for their electrical properties [43]. It was observed that additions of 75–85 w% of vanadium can react with various metaphosphates (Ba, Pb, Li, Na, Cd, V, and K) to generate good quality glasses with reproducible properties. The electrical resistance of these glasses depends in particular on their thermal history below the liquidus point and above the glass transition point. When the glasses are heat-treated and devitrification occurs, there is a sudden change in their resistivity. For example, for a glass containing potassium metaphosphate, the resistivity is reduced from 10^3^ to 10 ohm.cm. Likewise, the introduction of Nb_2_O_5_ and V_2_O_5_ as dopants has been analyzed in the field of glass thin film deposition for the production of hybrid microelectronic systems with oscillation, amplification, memory or current control (thyristors) properties [44].

Samples doped with 3 w% Nb_2_O_5_ and 3, 5, 12 and 20 w% V_2_O_5_ introduced prior to the addition of TEOS (in the form of NbCl_5_ and VOSO_4_.5H_2_O, respectively), were prepared in the polyphasic processing of the SG4 glaze (Fig. 2).

**Fig. 7:**
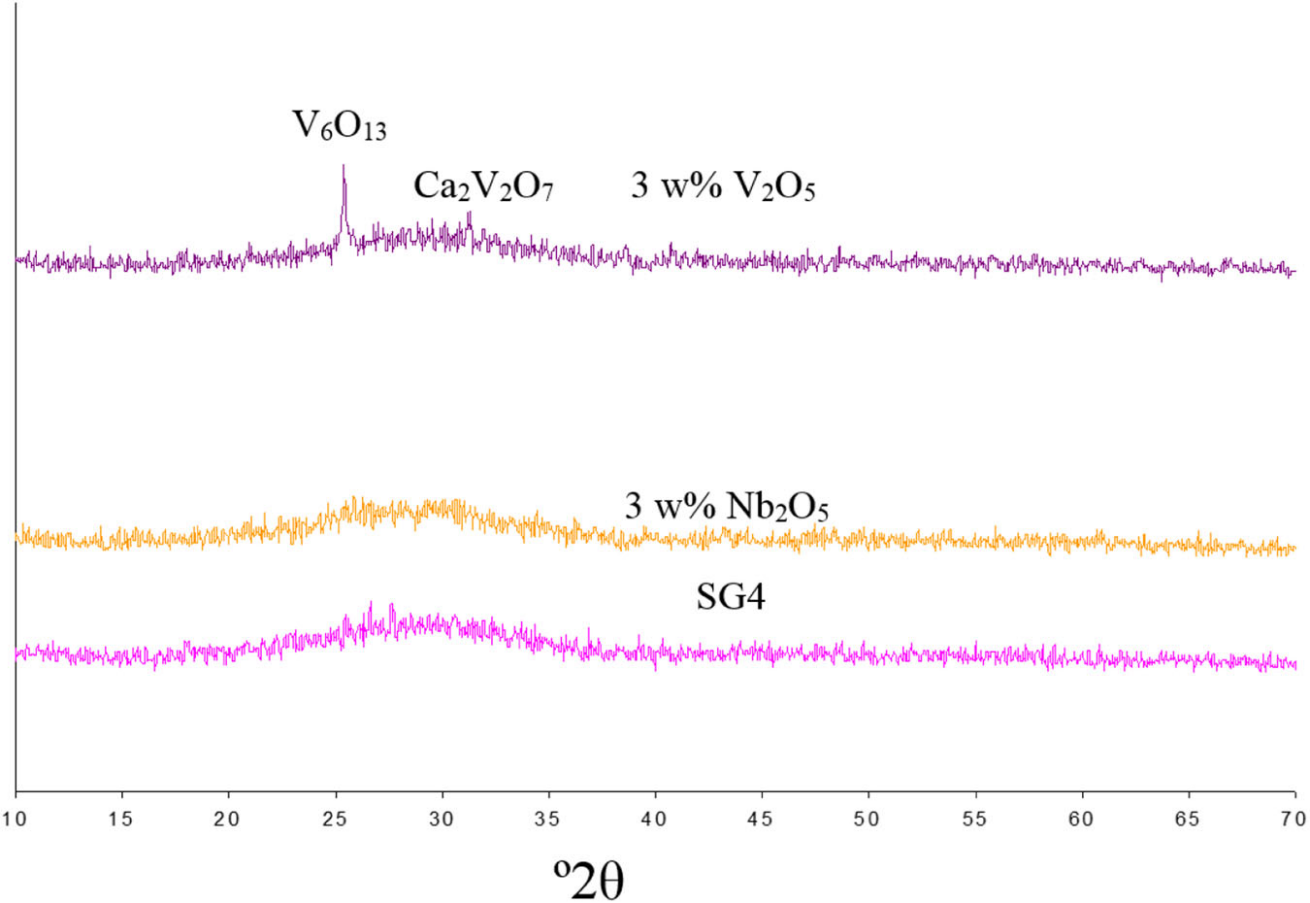
XRD of SG4 and its 3 w% modification with Nb_2_O_5_ (SG4Nb) and 3 w% of V_2_O_5_ (SG4V).

**Fig. 8:**
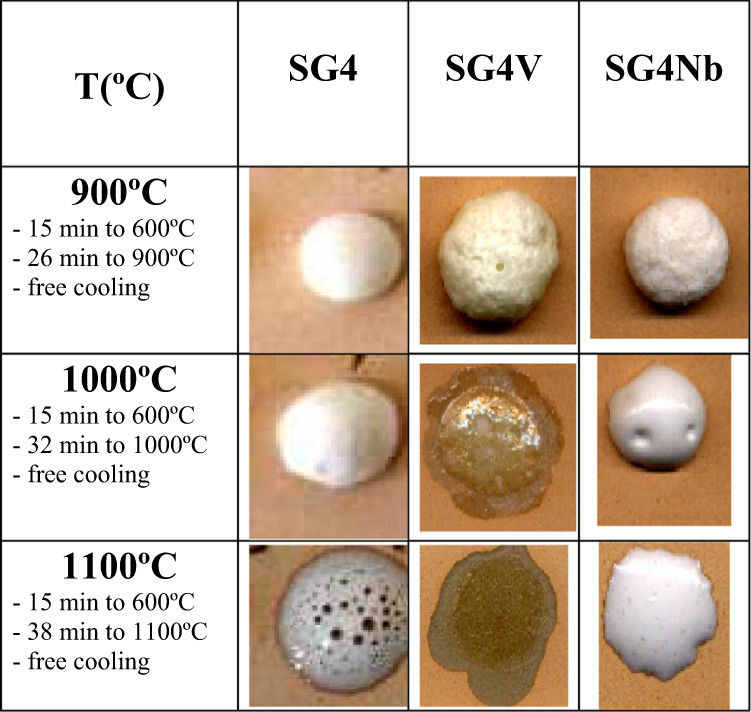
Evolution of the fusion buttons with temperature of the stabilized materials at 600 °C.

The Nb-doped glaze remains vitreous with a X-ray diffractogram (Fig. 7) similar to SG4. In contrast, the V-doped glaze exhibits diffraction peaks associated with Ca_2_V_2_O_7_ and V_6_O_13_ oxides. Figure 8 shows the evolution of the corresponding melting buttons; it is observed that the sample with vanadium degasses at 900 °C and melts with low surface tension at 1000 °C. On the other hand, the sample with Nb is an opaque white glaze with improved performance compared to SG4.

From the perspective of the photocatalytic activity of the glazes, Nb does not modify the SG4 band gap, whereas vanadium decreases it significantly (SG4 and SG4Nb, 3.8 eV; SG4V, 3.2 eV, Table 2). Despite this, vanadium maintains the levels of photoactivity of SG4 (t_1/2_ = 84 min), on the other hand, doping with Nb induces the loss of activity (t_1/2_ similar to control test). The role of Nb^5+^ ion as a glass-network former in NbO_6_-octahedra [48] in contrast to vanadium ions acting as glass modifier in the 5+ and 4+ oxidation states in fourfold coordination in silicate glasses [49], can explain the stabilization effect of niobium on the glassy network that facilitates the recombination of electron-hole pairs and therefore the loss of photocatalytic activity.

**Table 2:** Comparison of glazes doped with Nb and V and kinetic results in Orange II photodegradation test

Sample	Doping	E_g_ (eV)	Langmuir-Hinshelwood Kinetic	
			t_1/2_ (min.)	R^2^
Anatase	---------	3.0 (I)	42	0.991
SG4	----------	3.8 (D)	77	0.995
SG4V	3 w% V_2_O_5_	3.2 (D)	84	0.993
SG4Nb	3 w% Nb_2_O_5_	3.8 (D)	152	0.997
CONTROL	------------	------	151	0.993
SG4V3	3 w% V_2_O_5_	3.2 (I)	84	0.995
SGV45	5 w% V_2_O_5_	2.2 (I)	55	0.997
SGV412	12 w% V_2_O_5_	2.5 (I)	70	0.998
SGV420	20 w% V_2_O_5_	2.0 (I)	82	0.997

**Fig. 9:**
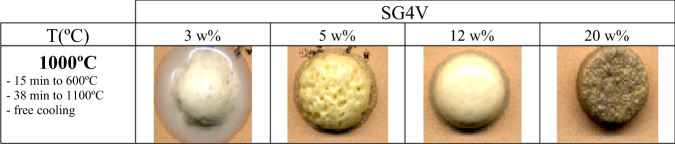
Evolution of melting buttons at 1000 °C of vanadium-modified glazes.

Given that in the literature [43] the electrical properties of vanadium-doped glasses show changes in the case of high vanadium concentrations, samples with vanadium content of up to 20 w% were prepared. The corresponding melt buttons prepared and fired at 1000 °C (see Fig. 9), show a yellowish hue from the sample with 5 w% of vanadium and brownish with 20 w% addition with poorly resolved vitrification. The fusibility decreases with the increase of vanadium in the sample, presenting a better performance in the sample with 3 w% of vanadium, although in the samples with 5 and 12 w% the characteristics of the SG4 glaze are largely preserved.

The X-ray diffraction of glazes shows the devitrification of the Ca_2_V_2_O_7_ and V_6_O_13_ phases discussed above. The intensity of the corresponding diffraction peaks increases slightly with the vanadium content, although anomalously this intensity is very low in the 5 w% sample.

**Fig. 10:**
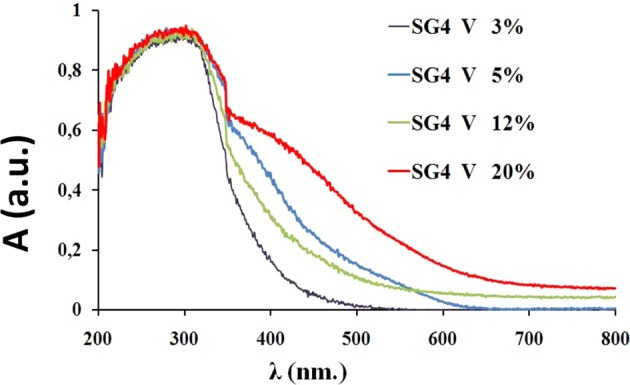
UV-Vis-NIR spectra of vanadium-modified glazes.

The UV-Vis-NIR spectra are shown in Fig. 10. They show a broad band associated with charge transfer centered around 300 nm of the SG4 glaze. The addition of vanadium induces the growth of another charge transfer band that grows towards the visible region with the increase of vanadium in the sample. As a result of this, the band gap values of the material decrease significantly with increasing vanadium, as shown in Table 2.

The rate of degradation of Orange II with increasing V (Table 2) presents an abnormally high value of t_1/2_ for the 3 w% sample, with the half-life period decreasing sharply in the 5 w% sample and subsequently increasing with increasing vanadium content. This anomaly was also observed in the evolution of X-ray diffractograms and in the band gap; the 5 w% shows an abnormally low band gap value (the band gap changes in a similar way to that of the photocatalytic activity). The low crystallization of Ca_2_V_2_O_7_ (triclinic, *SG P-1*) and V_6_O_13_ (monoclinic, *SG C2/m*) in the 5 w% sample indicates that vanadium incorporated into the glassy lattice shows a better catalytic activity associated with the low half-life period of Orange II degradation of the sample, despite the fact that the Ca_2_V_2_O_7_ [50] and particularly species with mixed oxidation states (V^4+^ or V^5+^) as in V_6_O_13_ are considered photocatalytically active sites [51]. On the other hand, the yellow color of the samples associated with the low bandgap of around 2–2.5 indicates that these vanadium doped samples efficiently absorb visible light and the catalytic process would be driven by visible light irradiation at room temperature as Zavahir et al. [51] described for V_6_O_13_.

In summary, doping with niobium does not induce significant modifications in the SG4 band gap and significantly degrades the photocatalytic activity. In contrast, vanadium doping produces a strong decrease in the band gap and a substantial improvement in photoactivity in the 5 w% addition sample, associated with good vitrification.

#### Effect of the addition of SnO_2_ on the photoactivity of the SG4 glaze

**Fig. 11:**
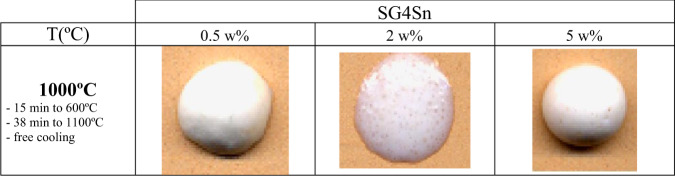
Evolution of melting buttons at 1000 °C of tin-modified SG4 glazes.

Samples modified with 0.5, 2 and 5 w% SnO_2_ introduced as final addition before TEOS in the form of SnCl_2_·2H_2_O were also prepared in the polyphasic processing of the SG4 glaze (Fig. 2). The evolution of the melting buttons produced from the stabilized gels as a function of the increase in the tin content of the samples (Fig. 11), shows a good vitrification of the stabilized gels at 1000 °C with an increase in whiteness and opacity of the glaze with respect to SG4. However, the sample with 2 w% SnO_2_ exhibited degassing.

**Fig. 12:**
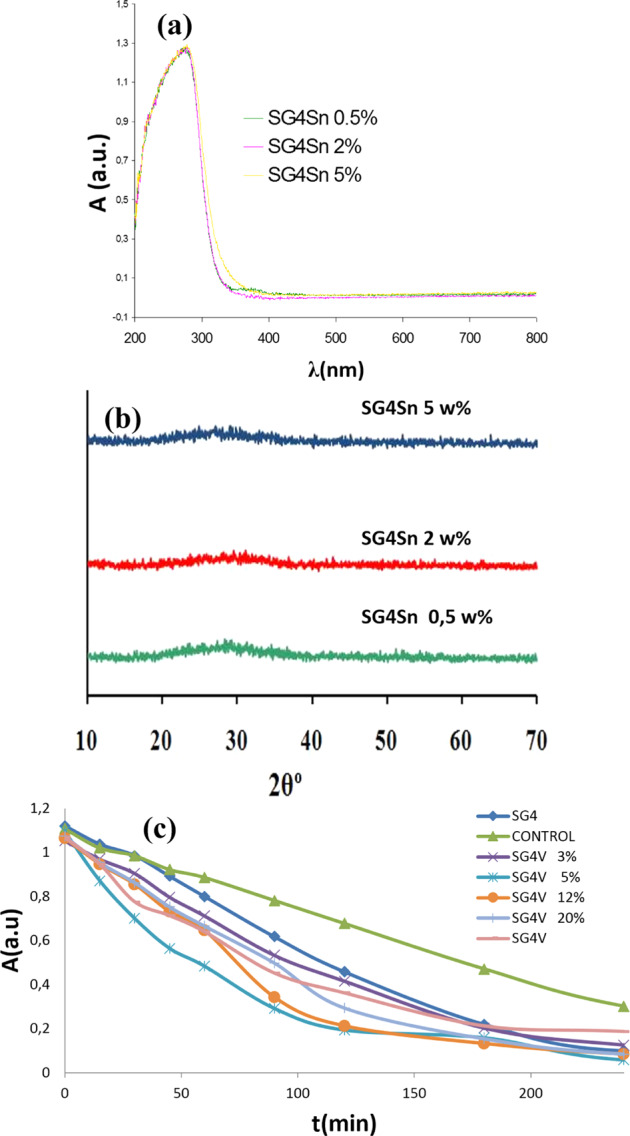
Characteristics of the SG4 glaze modified with tin oxide (**a**) UV-Vis-NIR diffuse reflectance spectra of the powders; (**b**) X-ray diffraction of the samples; (**c**) Orange II photodegradation test.

Figure 12 shows the characteristics of the tin-modified SG4 glaze samples. Figure 12a shows the UV-Vis-NIR spectra of the powders with different additions of tin oxide. It is observed that tin oxide does not significantly modify the charge transfer band of the semiconductor and the band gap of the parent glaze, with only the 5 w% sample exhibiting a slight increase in the width of the forbidden band. Figure 12b shows the X-ray diffraction of the samples, which indicates good vitrification without the appearance of crystallized phases. Figure 12c shows the Orange II photodegradation test indicating a moderate photocatalytic activity of the samples. Table 3 shows the Langmuir-Hinshelwood kinetic model results from the photodegradation on Orange II, showing a similar photocatalytic activity to the parent glaze in the additions of 0.5 and 2 w% of tin oxide and an enhanced photoactivity in the 5 w% sample (t_1/2_ = 58 min).

**Table 3:** Characteristics of the tin oxide modified SG4 glaze samples and data from the Langmuir-Hinshelwood kinetic model in photodegradation test on Orange II (D: direct semiconductor, I: indirect semiconductor)

SnO_2_ (w%)	E_g_ (eV)	Langmuir-Hinshelwood Kinetic	
		t_1/2_ (min.)	R^2^
anatase	3.0 (I)	42	0.991
SG4	3.8 (D)	77	0.995
0.5	3.8 (D)	77	0.993
2	3.8 (D)	75	0.995
5	3.7 (D)	58	0.996
CONTROL	------	151	0.993

### Effect of devitrification agents (ZrO_2_ to crystallize zircon, Al_2_O_3_ to anorthite, Mo_2_O_3_ to powellite and ZnO to gahnite ZnAl_2_O_4_.)

The induction of crystallization within a glaze when it is fired (devitrification) can be an interesting alternative to modify its properties. In this sense, glass-forming elements that present high ionic potentials such as Ti, Zr, Sn or Al, will tend to form MO_n_ structural units given their relative acidity, thus forming nanocrystals within the vitreous network. Specific examples of such dopants include Zn (which forms willemite or gahnite); Ti (which forms rutile, anatase or sphene); Zr (which forms zirconia and zirconium silicate (zircon)); Sn (which forms cassiterite or tin sphene); and Al (which forms corundum, gahnite, anorthite, gehlenite, spodumene or celsian). Calcium dopants also devitrify under certain conditions to form a wide group of crystalline phases such as wollastonite, diopside, scheelite or powellite [52, 53]. In this section, the study of the photocatalytic activity of the SG4 glaze modified by addition of one of the aforementioned devitrification ions is described and the vitreous powders and also the glazes fired on a ceramic substrate that devitrify the phase have been analyzed:

- 3 w% ZrO_2_ as zirconium acetate Zr(CH_3_COO)_4_ inducing the possible devitrification of zirconia and/or zirconium silicate.
- 10 w% Al_2_O_3_ as aluminum nitrate Al(NO_3_)_3_.9H_2_O inducing the devitrification of anorthite CaAl_2_Si_2_O_8_.
- 3 w% Mo_2_O_3_ as ammonium molybdate (NH_4_)_6_Mo_7_O_24_.4H_2_O inducing the crystallization of powellite CaMoO_4_.
- 10 w% ZnO as zinc nitrate Zn(NO_3_)_2_.6H_2_O inducing crystallization of gahnite ZnAl_2_O_4_.

**Fig. 13:**
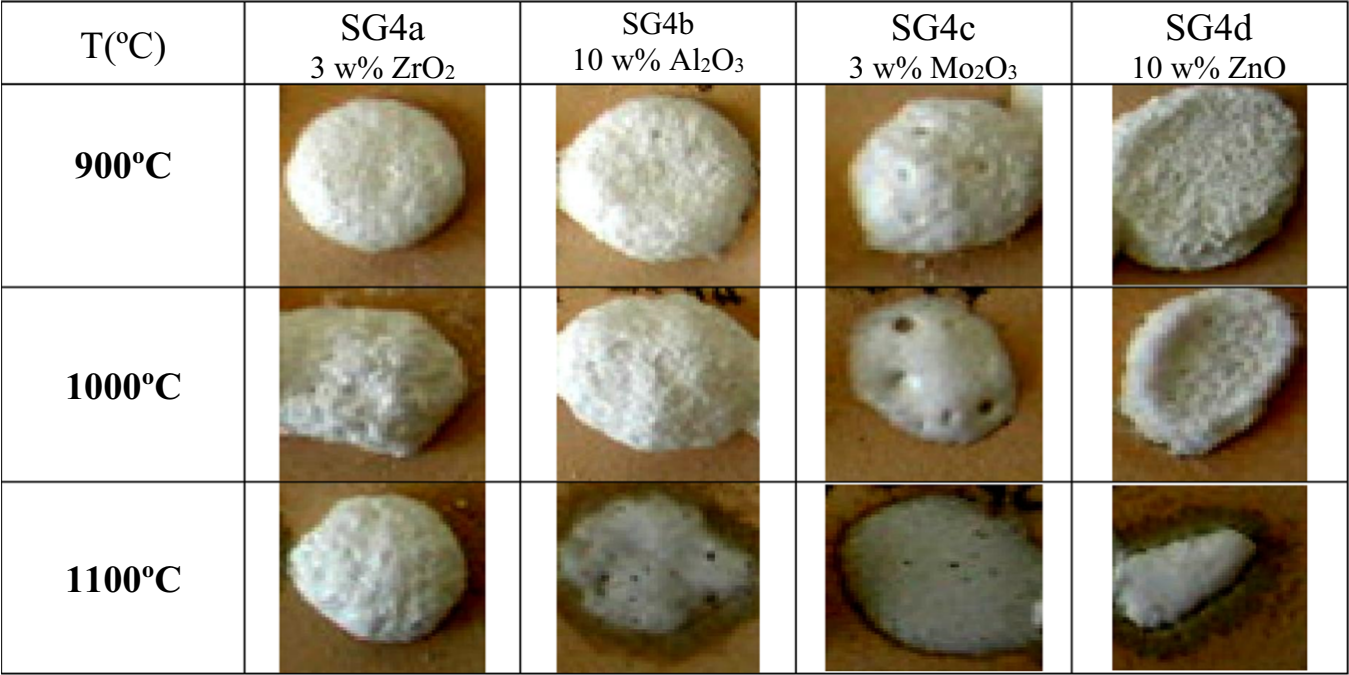
Evolution of the fusion buttons with temperature of monophasic xerogels stabilized at 600 °C.

**Fig. 14:**
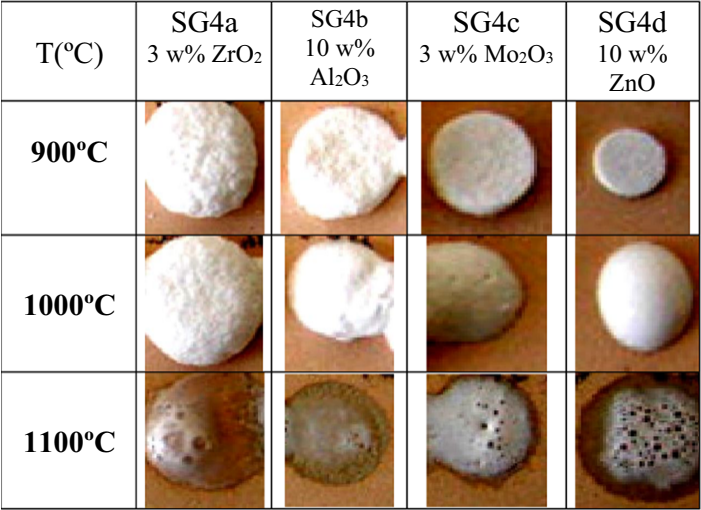
Evolution of the fusion buttons with temperature of polyphasic xerogels stabilized at 600 °C.

The samples were prepared according to the mono and polyphasic processes for multicomponent glazes (Fig. 2), adding the reagents before introducing the tetraethylorthosilicate TEOS into the mixture. Figure 13 and 14 show the fusion buttons prepared from the monophasic and polyphasic gels, respectively; the additives increase the opacity and whiteness of the fired glazes. Among the different additives, the lowest fusibility occurs for the addition of zirconium oxide or alumina. A high degree of degassing is observed in all samples.

**Fig. 15:**
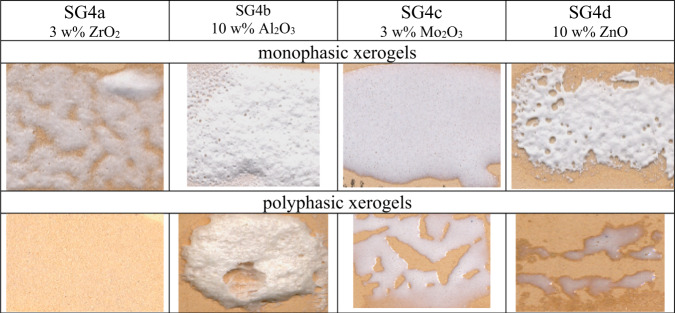
Doctor Blade depositions of the monophasic and polyphasic xerogels fired with a single fire cycle.

Figure 15 shows the Doctor Blade depositions of the monophasic and polyphasic xerogels fired using a single firing cycle. Adequate coverage is observed in the case of zirconium oxide and molybdenum oxide additions; in the case of zinc and aluminum additions the glaze is less fusible in the “monoporosa” single fire cycle.

The difficulty in generating homogeneous glaze films ruled out the samples modified with zinc and aluminum for the subsequent photochemical study.

**Fig. 16:**
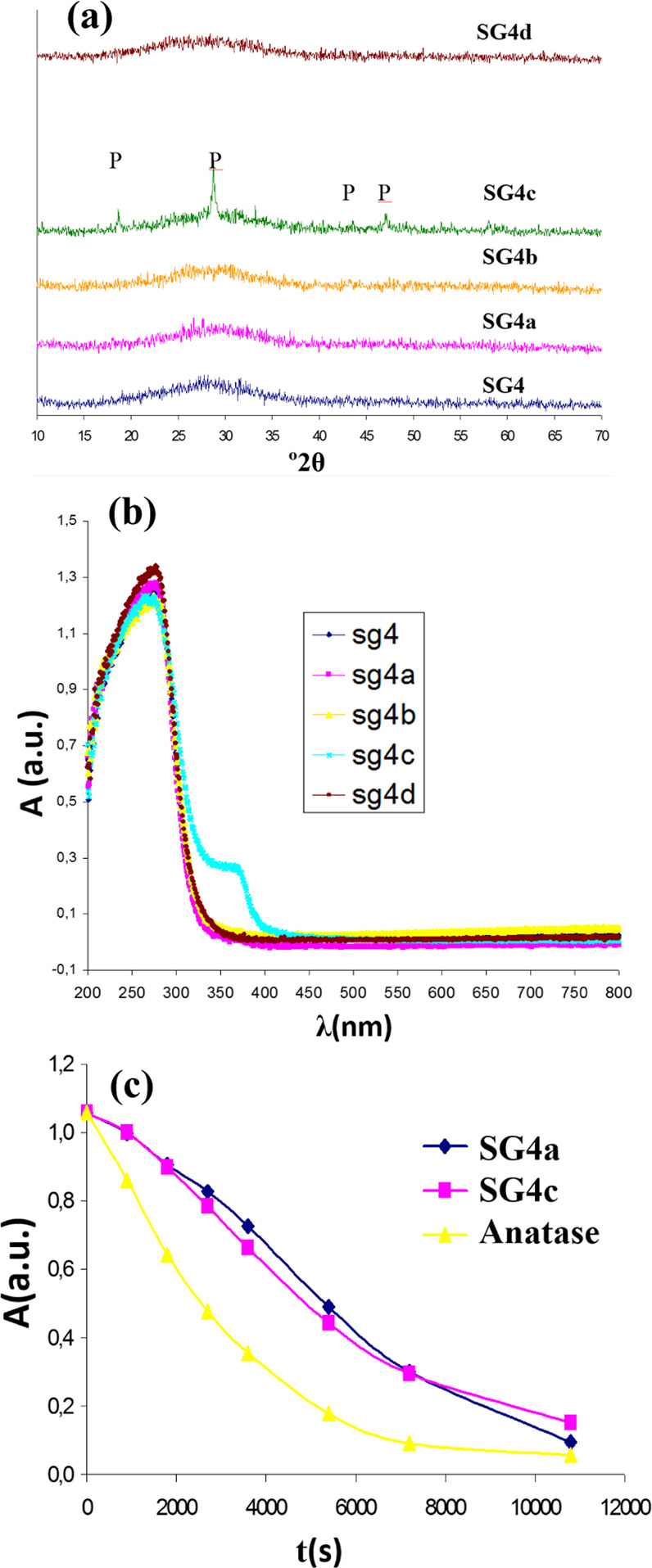
Characteristics of the SG4 glaze sample modified with devitrification agents: (**a**) UV-Vis-NIR spectra of powder samples (**b**) X-ray diffraction of the samples (P = powellite CaMoO_4_); (**c**) Orange II photodegradation test.

Figure 16 shows the characteristics of the SG4 glaze sample modified with the devitrification agents. Figure 16a shows the X-ray diffraction of the samples indicating good vitrification of the samples without the appearance of crystallized phases, except in SG4c that devitrifies to form powellite. Figure 16b shows the UV-Vis-NIR spectra of the modified powders with the devitrification agents. It is observed that all samples present similar charge transfer band positions, except for the glaze modified with ammonium molybdate SG4c, which presents an additional band of lower intensity whose threshold wavelength reduces the band gap of the material. Figure 16c illustrates the Orange II photodegradation tests, indicating that the photocatalytic activity is lower than the reference anatase and similar or lower than the SG4 glaze, as indicated by the half-live obtained by fitting the photodegradation curve according to the Langmuir-Hinshelwood model in Table 4.

**Table 4:** Band gap of SG4 glaze sample modified with devitrification agents and kinetic results in Orange II test (D: direct semiconductor, I: indirect semiconductor)

Sample	additive	Eg (eV)	Langmuir-Hinshelwood kinetics	
			t_1/2_ (min.)	R^2^
Anatase	---------	3.0 (I)	42	0.991
SG4		3.8 (D)	77	0.995
SG4a	3 w% ZrO_2_	3.8 (D)	105	0.995
SG4c	3 w% Mo_2_O_3_	3.2 (D)	76	0.997
CONTROL	------------	------	151	0.993

**Fig. 17:**
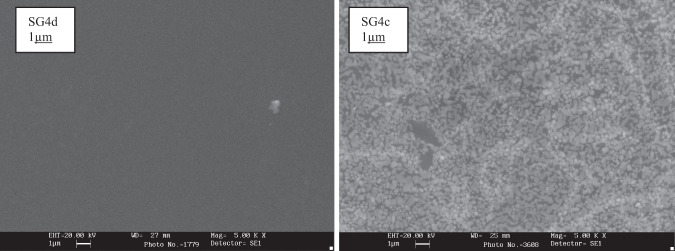
SEM micrographs of the Doctor Blade depositions of the polyphasic xerogels fired with a single fire cycle of SG4d and SG4c samples.

Finally, Fig. 17 shows the SEM micrographs of the Doctor Blade depositions of the polyphasic SG4d and SG4c xerogels fired with a single firing cycle, showing the bulk crystallization of cubic powellite CaMoO_4_ in the case of SG4c and the absence of crystallization in the SG4d modified with ZnO (SG4a and SG4b show similar homogeneous microstructures).

## Conclusions

Multicomponent frits (zinc-potassium borosilicate system) with similar behavior to conventional ceramic frits for single-firing ceramic glazes (“monoporosa” glazes 1080 °C) were prepared by Sol-Gel methods (monophasic and polyphasic gels):

- The zinc and potassium borosilicate-based glaze, modified with alumina and calcium, is considered the optimum for conventional applications, and is obtained through the polyphasic process given its simplicity. This glaze, referred to as SG4, presents a moderate photoactivity with a half-life period evaluated according to the Langmuir-Hinshelwood model of 77 min.
- The effect of doping with bandgap modifiers (V_2_O_5_, Nb_2_O_5_ and SnO_2_) was analyzed. The modification with niobium does not change the band gap of the glaze (3.8 eV) but it reduces the photocatalytic activity. In contrast, vanadium doping decreases the band gap of the glaze (3.2 eV) and maintains the levels of photoactivity. Incorporations of vanadium in increasing amounts up to 20% as vanadium pentoxide indicate an optimal concentration of around 5% that decreases the half-life of photodegradation on Orange II to 55 min and is associated with good vitrification in the fusibility test using fusion buttons. Finally, addition of tin to the SG4 composition does not significantly modify the charge transfer band of the semiconductor and the bandgap of the parent glaze, with only a slight decrease in the width of the forbidden band being observed at a dopant level of 5 w%. X-ray diffraction analysis of the samples indicated good vitrification without the appearance of crystallized phases and photodegradation tests showed a moderate photocatalytic activity similar to the parent glaze with the additions of 0.5 and 2 w% of tin oxide and a good photocatalytic activity in the 5 w% sample (t_1/2_ = 58 min)
- The effect of doping with devitrification agents (ZrO_2_ to crystallize zircon; Al_2_O_3_ to crystallize anorthite; Mo_2_O_3_ to form powellite; and ZnO to form gahnite) were analyzed. The UV-Vis-NIR spectra of the modified powders show a similar charge transfer band position to that of the SG4 parent glaze, except for the glaze modified with ammonium molybdate, which presents an additional band of lower intensity whose threshold wavelength reduces the band gap. X-ray diffraction of the samples shows good vitrification of the samples without the appearance of crystallized phases except in SG4c that devitrifies to form powellite. In this case, the corresponding Orange II photodegradation test shows that the photocatalytic activity is lower than the reference anatase and similar or lower to that of the SG4 glaze.

## Data Availability

All data and materials are available.
